# Nonlinear Magnetoelectric Response of Fe_73.5_Cu_1_Nb_3_Si_13.5_B_9_/Piezofiber Composite for a Pulsed Magnetic Field Sensor

**DOI:** 10.3390/ma12182866

**Published:** 2019-09-05

**Authors:** Caijiang Lu, Hai Zhou, Aichao Yang, Zhengyu Ou, Feihu Yu, Hongli Gao

**Affiliations:** 1 Department of Electromechanical Measuring and Controlling, School of Mechanical Engineering, Southwest Jiaotong University, Chengdu 610031, China (H.Z.) (Z.O.) (F.Y.) (H.G.); 2 Jiangxi Electric Power Research Institute, Nanchang 330096, China

**Keywords:** magnetoelectric response, pulsed magnetic field sensor, piezofiber, composite

## Abstract

In this paper, we report the nonlinear magnetoelectric response in a homogenous magnetostrictive/piezoelectric laminate material. The proposed magnetoelectric stack Fe_73.5_Cu_1_Nb_3_Si_13.5_B_9_/piezofiber is made up of high-permeability magnetostrictive Fe_73.5_Cu_1_Nb_3_Si_13.5_B_9_ foils and a piezoelectric Pb(Zr, Ti)O_3_ fiber composite. The time dependence of magnetoelectric interactions in the Fe_73.5_Cu_1_Nb_3_Si_13.5_B_9_/piezofiber structure driven by pulsed magnetic field was investigated in detail. The experimental results show that the magnetoelectric effect is strongly dependent on the external bias magnetic and pulsed magnetic field parameters. To detect the amplitude of a pulsed magnetic field, the output sensitivity reaches 17 mV/Oe, which is excited by a 100 μs width field. In addition, to measure the pulsed width, the output sensitivity reaches 5.4 mV/μs in the range of 0–300 μs. The results show that the proposed Fe_73.5_Cu_1_Nb_3_Si_13.5_B_9_/piezofiber sensor is ideally suited for pulsed magnetic field measurement.

## 1. Introduction

Magnetic field sensors are widely used in various fields, for example, in the industry, agriculture, medicine, aerospace, marine, exploration, and drilling. In recent years, the magnetoelectric (ME) composites with a product property of magnetostrictive and piezoelectric phases have been of great importance for the realization of a magnetic sensor [1,2,3]. In the ME effect, an electric field is induced under the application of a magnetic field or conversely, magnetization is induced under the application of an external electric field [1,2,3]. To date, different magnetic sensors based on ME composites have been experimentally and theoretically investigated in order to obtain the best ME coupling through changing the structures [4,5,6,7], materials [8,9,10], number of layers [11,12], etc.

Among the proposed composites, the 2-2 laminated composite material has a good ME coupling effect at room temperature, a large degree of freedom in design, and a strong application prospect, which provides a clear physical meaning for the design of a new generation of electronic devices [1,2,3,4,5,6,7,8,9,10,11,12]. Recently, for static or quasi-static magnetic field sensing in an unshielded room temperature and pressure and lab environment, a high direct current (DC) magnetic field sensitivity of 2.8 Hz/nT and a limit of detection of 800 pT were reported in the NEMS AlN/FeGaB resonator [13]. For alternating current (AC) magnetic field sensing, an extremely low equivalent magnetic noise of 5.1 pT/√Hz at 1 Hz was reported in the Metglas/piezofiber structure [14]. A super-high magnetic sensitivity of 1.35 × 10^−13^ T was directly detected at 23.23 kHz in the 1D (1-1) connectivity ME composites of Metglas/PMN-PT [15].

In practice, transient pulsed magnetic field measurement exists in many fields, such as lightning current in power systems [16,17] and aircrafts [18,19], crack detection with pulsed magnetic flux leakage techniques [20,21], and transient electromagnetic measurement in transformer substations [22]. However, to date, most of the reported theoretical and experimental studies carried out on ME sensors have been investigated under the premise of static magnetic field and standard sine wave magnetic field excitation [1,2,3,4,5,6,7,8,9,10,11,12,13,14,15]. Only a few studies have focused on the transient response of ME materials in pulsed magnetic fields [23]. The magnetic–mechanical–electric nonlinear coupling mechanism of the magnetostrictive/piezoelectric structure should be different by changing the parameters of transient magnetic fields. Thus, for potential applications in pulsed magnetic field measurement of the ME composite, this paper focused on the transient nonlinear ME response under a pulsed magnetic field. The influence of the pulsed magnetic field parameters and external bias field on the ME structure were investigated in detail.

## 2. Experimental

**Figure 1:**
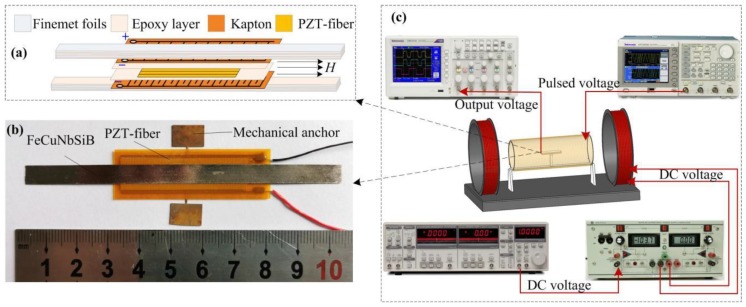
(**a**) Schematic illustrations of the FeCuNbSiB/piezofiber magnetoelectric (ME) composites, (**b**) photograph of the FeCuNbSiB/piezofiber ME composites, (**c**) schematic illustrations of the experimental setup.

The ME structure consists of Fe_73.5_Cu_1_Nb_3_Si_13.5_B_9_ (FeCuNbSiB) and piezofiber, as shown in Figure 1a,b. The FeCuNbSiB (International standard trademark 1K107, produced by Foshan Huaxin Microlite Metal Co., Ltd., Foshan, China) is a pizeomagnetic phase with a high permeability (μr = 30,000), high saturation magnetization (μ_0_Ms = 1.45 T), and a large anisotropic constant (−30,000 J/m^3^). The dimensions of the FeCuNbSiB foils are 10 × 6 × 0.025 mm^3^. The Pb(Zr, Ti)O_3_ fiber composite (piezofiber) (M4010-P1, provided by Smart Material Cor., Sarasota, FL, U.S.A.) is a piezoelectric phase consisting of rectangular piezoceramic rods (40 mm long and 180 μm thick) sandwiched between layers of adhesive, electrodes, and polyimide film. The electrodes are attached to the film in an interdigitated pattern which transfers the applied voltage directly to and from the ribbon-shaped rods.

As shown in Figure 1a, two FeCuNbSiB layers (each made by three FeCuNbSiB foils) were subsequently laminated to both the top and bottom surfaces of the Pb(Zr, Ti)O_3_ (PZT)-fiber layer. Under an AC magnetic field, a mechanical strain was generated in the FeCuNbSiB layer and was then transferred to the piezofiber, resulting in the generation of charges due to the direct piezoelectric effect. As shown in Figure 1b, the ME FeCuNbSiB/piezofiber was designed to operate as a half-wavelength longitudinal resonator vibrating freely at both ends. Thus, one mechanical anchor (made by Beryllium bronze plate, produced by Shanghai Dayu Metal Products Co., Ltd., Shanghai, China) bonded with epoxy at the middle of the FeCuNbSiB/piezofiber where the displacement was zero.

The experimental setup is shown in Figure 1c. A signal generator (Tektronix AFG3021B, Tektronix Inc., Beaverton, OR, USA) provided a controllable input current to a long straight solenoid coil with 1800 turns and a 182 mm length, which was used to provide the pulsed magnetic field. A Helmholtz coil (Linkphysics Co., Ltd., Shanghai, China) was used to provide the DC bias magnetic field, which was driven by a power amplifier (KEPCO Bipolar Operational Power Supply, KEPCO Inc., Flushing, NY, USA.). The ME output voltages of the FeCuNbSiB/piezofiber laminate were measured with a lock-in amplifier (SR-830, SRS, Sunnyvale, CA, USA.) and an oscilloscope (Tektronix Inc., Beaverton, OR, USA). The bias magnetic field H_bias_ was measured with a Gauss meter. The AC magnetic field was calculated in the experiments. The resistance of the long straight solenoid coil was measured as 37.8 Ω by a multimeter (Fluke Corporation, Everett, WA, USA). In addition, the voltage of the long straight solenoid in the experiments was measured by an oscilloscope. Thus, the actual current *I* in the long straight solenoid can be calculated as I = voltage/37.8. Then, the AC magnetic field of the central solenoid H = n*I (A/m), where n = 1800/0.182. Before the experiments, the calculated AC magnetic field was calibrated by a Fluxgate probe (CH-Magnetoelectricity Technology, Beijing, China).

## 3. Results and Discussion

**Figure 2:**
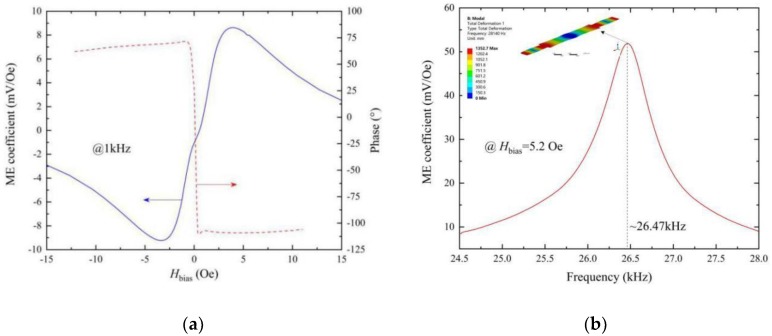
The ME voltage coefficient of the FeCuNbSiB/piezofiber laminates: (**a**) ME coefficient α_ME_ as a function of the bias magnetic field H_bias_ in response to a ~1 kHz sine driving magnetic field, and (**b**) α_ME_ as a function of the AC sine magnetic drive frequency sweeping through the electromechanical resonance.

The ME response of the FeCuNbSiB/piezofiber composite was calibration-tested under a standard sine magnetic field. Figure 2a shows the ME coefficient α_ME_ and the phase angle as a function of the bias magnetic field H_bias_ driven at a 1 kHz sine magnetic field. As shown in Figure 2a, α_ME_ increased with increasing H_bias_ up to about H_bias_ = 3.6 Oe reached a maximum value of α_ME_ = 9.2 mV/Oe and then gradually decreased as H_bias_ was further increased. The induced voltage was independent of H_bias_ history and no offset value was found near H_bias_ = 0 Oe. These characteristics are quite important to magnetic field detection. In addition, as the direction of H_bias_ was changed, a 180° phase shift was found, as shown in Figure 2a. Next, α_ME_ was measured as a function of the AC magnetic field frequency at H_bias_ = 5.2 Oe while sweeping near the mechanical resonance, as shown in Figure 2b. As this figure shows, the fundamental resonant frequency for the FeCuNbSiB/piezofiber composite was ~26.47 kHz. At this resonant frequency, a value of α_ME_ > 50 mV/Oe was reached. The natural period of the FeCuNbSiB/piezofiber composite *T* = 1/*f*_r_ = ~37.78 μs.

For the ME composite consisting of mechanically coupled magnetostrictive and piezoelectric layers, the resonance frequency of the ME composite is [12]

(1) $$f_{r}=\frac{1}{2l}\sqrt{\frac{1}{\rho_{}s_{}^{}}}$$

where *l* is the length, and ρ and s are the average density and the equivalent elastic compliance, respectively. The length of the PZT-fiber is 40 mm. The $\rho_{p}$ and $s_{11}^{E}$ of PZT-fiber is 5.44 g/cm^3^ and 32.96 × 10^−12^ m^2^/N, respectively. The length of the FeCuNbSiB foils is 100 mm. The $\rho_{m}$ and $s_{33,m}^{H}$ of FeCuNbSiB is 7.25 g/cm^3^ and 5.2 × 10^−12^ m^2^/N, respectively. The calculated first-order longitudinal resonant frequencies of the PZT-fiber and the FeCuNbSiB foil are ~29.5 kHz and ~25.8 kHz, respectively. Thus, the resonant frequency for the FeCuNbSiB/PZT-fiber stack should be about ~25.8 kHz to ~29.5 kHz. In order to calculate the resonance frequency of FeCuNbSiB/piezofiber, we used the ANSYS 19.0 software (ANSYS, Inc., Canonsburg, PA, USA). In the simulations, the elastic modulus and density of the epoxy layer were 3 GPa and 5 g/cm^3^, respectively. The simulation results of the first-order longitudinal vibration are shown in the inset of Figure 2b. The first-order longitudinal resonant frequency of the FeCuNbSiB/PZT-fiber was ~28.14 kHz. The theory calculated and the simulation results agree with the experimental results.

**Figure 3:**
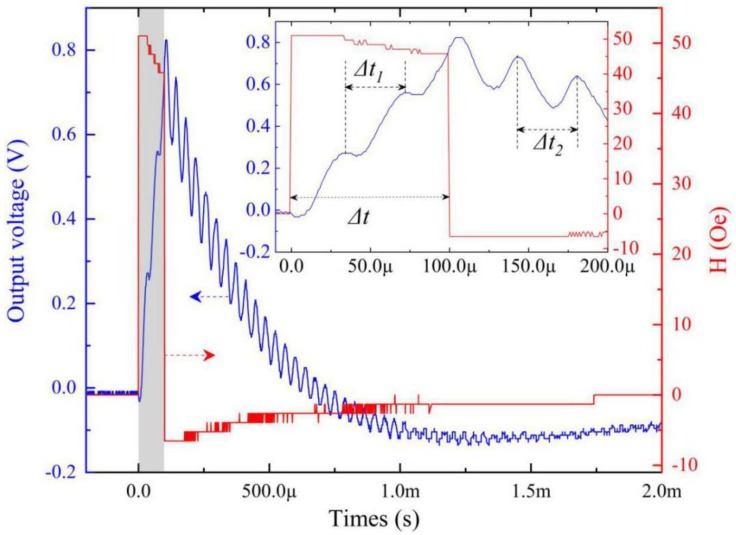
Time dependence output voltage *V*_o_ of the FeCuNbSiB/piezofiber structure driven by the magnetic field pulse with a width (Δt) of ~100 μs and an amplitude of 51 Oe. The inset shows the detail when times were 0–200 μs. The rise time of the pulse magnetic was ~8.4 ns. The bias magnetic field was set to zero.

Then, the transient nonlinear ME coupling was investigated in detail. Figure 3 shows the time dependence output voltage *V*_o_ of the FeCuNbSiB/piezofiber structure driven by the magnetic field pulse with a width (Δt) of ~100 μs and an amplitude of 51 Oe. The inset of Figure 3 shows the detail when times were 0–200 μs. The rise time of the pulse magnetic was ~8.4 ns. From this figure, the *V*_o_ increased gradually when the pulsed magnetic field increased rapidly to an amplitude of 51 Oe and maintained ~100 μs. This is because the magnetic energy increased gradually when the pulsed field maintained the amplitude. It is clear that *V*_o_ oscillated at the period Δt_1_ = ~37.78 μs. After t > Δt = 100 μs, the pulse magnetic field decreased rapidly to zero. However, the *V*_o_ decreased gradually and oscillated at the period Δt_2_ = ~37.78 μs. This is due to the inertia effect of the FeCuNbSiB/piezofiber structure. The FeCuNbSiB/piezofiber ME structure is a mechanical resonant structure that self-vibrates near the equilibrium position at the natural period *T* when the pulsed magnetic field vanishes. It is clear that the FeCuNbSiB/piezofiber structure always oscillates at the natural period *T* in the time domain. If the width of the pulsed magnetic field Δt is equal to *T* or multiples of *T*, what will happen?

**Figure 4:**
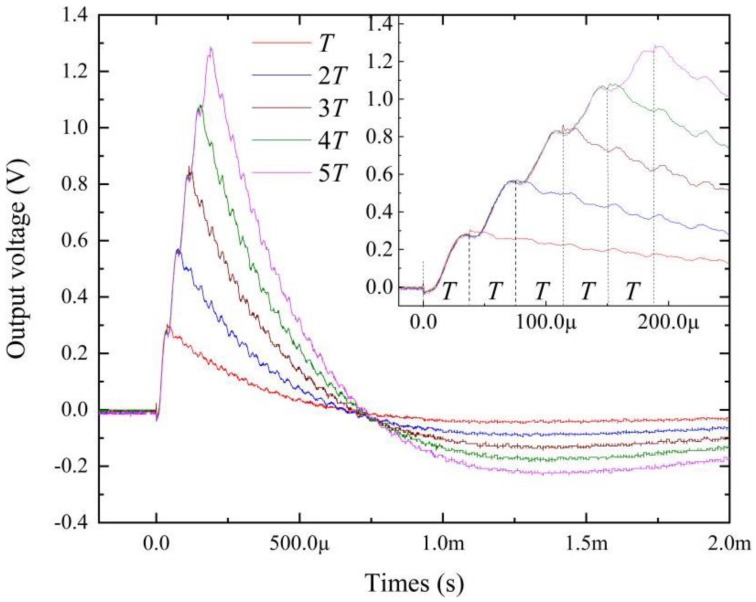
Time dependence output voltage *V*_o_ of the FeCuNbSiB/piezofiber structure driven by the magnetic field pulse with an amplitude of 51 Oe and a width of Δt = T–5T. The rise time of the pulse magnetic was ~8.4 ns. The bias magnetic field was set to zero.

Figure 4 shows the time dependence *V*_o_ of the FeCuNbSiB/piezofiber structure driven by magnetic field pulses with amplitudes of 51 Oe and widths of Δt = T–5T. The inset of Figure 4 shows the detail when times were 0–250 μs. After t >Δt, it is interesting that the self-vibration phenomena vanished when Δt = T–5T, which is extremely different from Figure 3. This result can be explained by the fact that the FeCuNbSiB/piezofiber structure was exactly in an equilibrium position when the pulse magnetic field vanished. Moreover, the other interesting result from Figure 4 is that *V*_o_ grew exponentially, which was excited by the same amplitude pulsed magnetic field of 51 Oe when Δt = T–5T. This result demonstrates that the ME FeCuNbSiB/piezofiber structure can be used as a pulse magnetic width measured device, and this will be investigated in detail in the following section.

**Figure 5:**
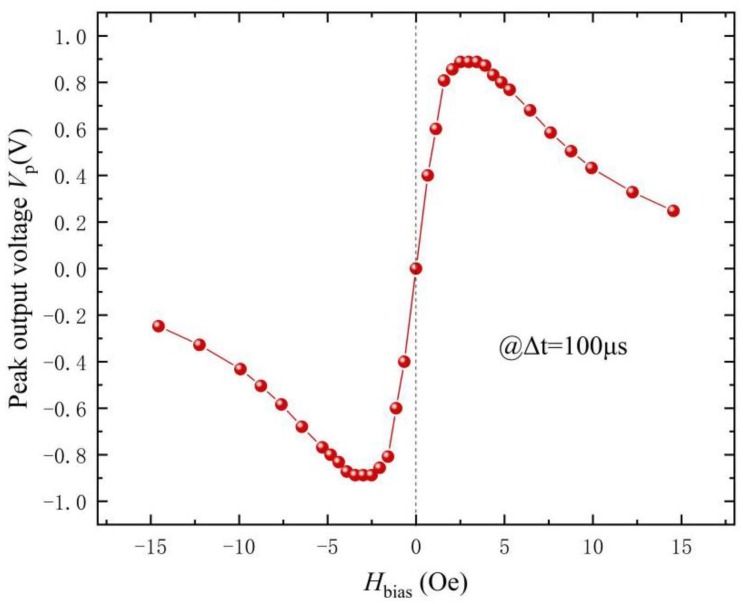
The peak output voltage *V*_p_ (maximum *V*_o_ in Figure 3) as a function of H_bias_ for the FeCuNbSiB/piezofiber structure driven by the pulsed magnetic field with Δt = ~100 μs.

Based on Figure 2a, the ME response of the FeCuNbSiB/piezofiber structure is dependent on the H_bias_. Therefore, the H_bias_ dependence of the ME response for the FeCuNbSiB/piezofiber structure driven by the pulsed magnetic field was also investigated. Figure 5 shows the peak output voltage V_p_ (maximum *V*_o_ in Figure 3) as a function of H_bias_ for the FeCuNbSiB/piezofiber structure driven by the pulsed magnetic field with Δt = ~100 μs. The curve tendency in this figure is the same as that in Figure 2a. The maximum V_p_ was ~0.9 V at H_bias_ = 3.6 Oe.

**Figure 6:**
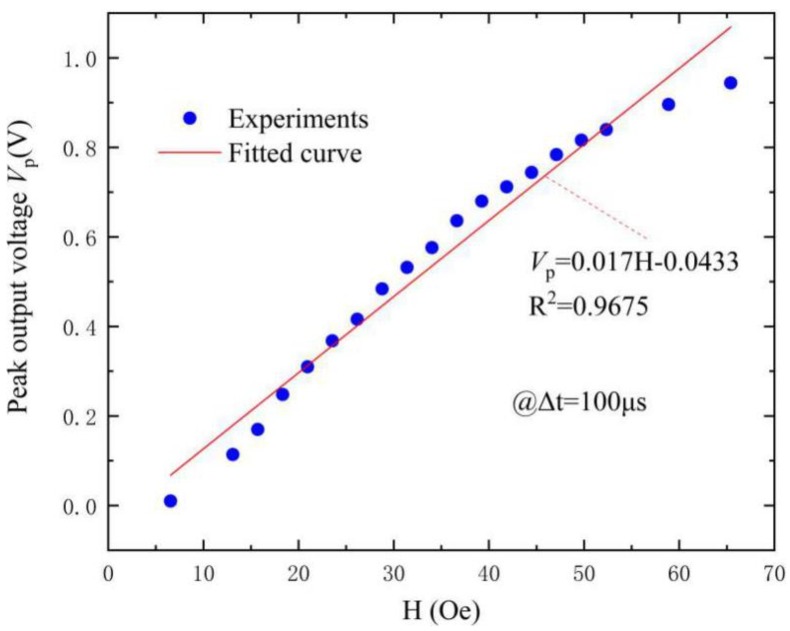
The peak output voltage *V*_p_ (maximum *V*_o_ in Figure 3) as a function of the amplitude of the pulsed magnetic field H_A_ for the FeCuNbSiB/piezofiber structure driven by the pulsed magnetic field with Δt = ~100 μs. The red line is the linear approximation.

Next, the transient sensing characteristics of the FeCuNbSiB/piezofiber structure was investigated. Firstly, the amplitude of the pulsed magnetic field H_A_ sensitivity was measured. Consequently, Figure 6 shows the peak output voltage (like the peak in Figure 3) as a function of H_A_ at H_bias_ = 0 Oe. The different values of amplitude of the pulsed magnetic field H_A_ were obtained at Δt = ~100 μs. The linear fitting expression based on the experimental data was *V*_p_ = 0.017*H*_A_–0.0433. Based on Figure 6, it is obvious that the induced ME voltage had a near linear (R^2^ = 0.9675) relation with H_A_. The observation indicates an improved detection sensitivity of 17 mV/Oe. Clearly, the presented FeCuNbSiB/piezofiber sensor seems to be an ideal application for the detection of amplitude variations of pulsed magnetic fields.

**Figure 7:**
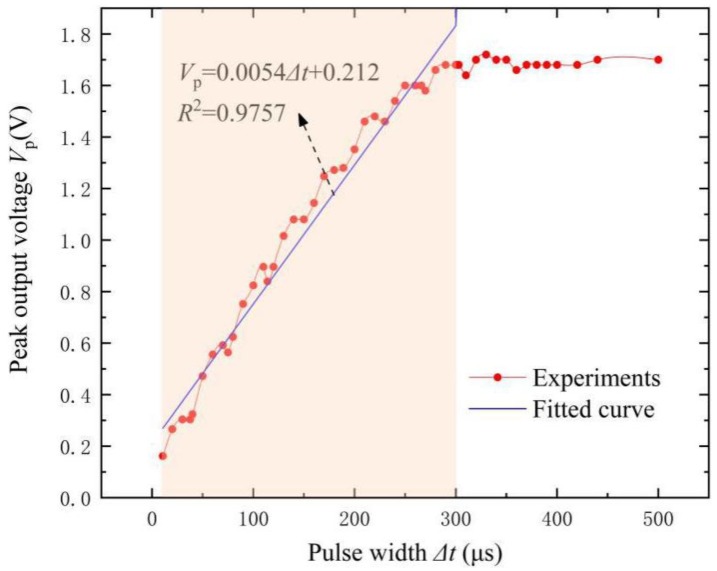
The peak output voltage *V*_p_ (maximum *V*_o_ in Figure 3) as a function of the width of the pulsed magnetic field Δt for the FeCuNbSiB/piezofiber structure at H_bias_ = 0 Oe. The blue line is the linear approximation. The amplitude of the pulsed magnetic field is 52.3 Oe.

From Figure 4, changing Δt results in the variation of the output voltage of the FeCuNbSiB/piezofiber structure. Figure 7 shows the peak output voltage (like the peak in Figure 3) as a function of the width of the pulsed magnetic field Δt = 0–500 μs as H_bias_ = 0 Oe. It is evident that the *V*_p_ increases with Δt and reaches its maximum value after Δt >300 μs. For the linear fitting at Δt = 0–300 μs, the relationship between *V*_p_ and Δt is *V*_p_ = 0.0054Δt + 0.212. One can see that the linear (R^2^ = 0.9757) dependence of the V_p_ on the Δt takes place in a region of 0 < Δt < 300 μs. Based on the slope of the plots, the sensitivity of the FeCuNbSiB/piezofiber structure for pulsed width sensing was determined to be 5.4 mV/μs. It can be concluded that this ME sensor is suitable for pulsed width measurement fields, such as lightning current monitoring.

## 4. Conclusions

In conclusion, a FeCuNbSiB/piezofiber structure prototype was fabricated and several experiments were conducted to illuminate the transient nonlinear ME response. The time dependence output voltage of the FeCuNbSiB/piezofiber structure was measured in the experiments, which showed that the FeCuNbSiB/piezofiber structure oscillated during the natural period T = ~37.78 μs in the time domain. Specifically, the self-vibration phenomena vanished after the excited field vanished when the width was equal to the multiples of the natural period T. The transient ME response was strongly dependent on the external bias magnetic field, which was the same as with the ME response driven by the sine magnetic field. The sensing characteristics to the pulsed magnetic field were also measured. The results show that the output sensitivities reached 17 mV/Oe Δt = 100 μs for detecting the amplitude of the pulse magnetic field and reached 5.4 mV/μs when Δt = 0–300 μs for detecting the pulsed width. The results obtained may be useful for the development of ME sensors for pulsed magnetic fields, such as the lighting current monitoring field.
